# Postoperative persistent diastolic dyssynchronous expansion in patients with Ebstein’s anomaly

**DOI:** 10.1371/journal.pone.0220890

**Published:** 2019-08-08

**Authors:** Kyung-Jin Kim, Kyung-Hwan Kim, Woong-Han Kim, Dae-Won Sohn

**Affiliations:** 1 Department of Internal Medicine, Division of Cardiology, Ewha Womans University Medical Center, Ewha Womans University School of Medicine, Seoul, Korea; 2 Department of Thoracic and Cardiovascular Surgery, Seoul National University Hospital, Seoul National University College of Medicine, Seoul, Korea; 3 Department of Internal Medicine, Cardiovascular Center, Seoul National University Hospital, Seoul National University College of Medicine, Seoul, Korea; Scuola Superiore Sant’Anna, ITALY

## Abstract

In Ebstein’s anomaly, maximal expansion in the atrialized right ventricle (RV) occurs during early diastole, whereas that of the functional RV occurs in late diastole, resulting in diastolic dyssynchronous expansion (DSE). We quantitatively assessed DSE and identified preoperative factors correlated with persistent DSE after surgery. Seventeen patients diagnosed with Ebstein’s anomaly in whom transthoracic echocardiography (TTE) and cardiac magnetic resonance (CMR) images available were retrospectively analyzed for quantitative DSE assessment and 10 patients who underwent surgery and postoperative TTE available were additionally analyzed for postoperative DSE. Severity of DSE was assessed by the time difference of maximal expansion between the atrialized and functional RV divided by the cardiac cycle length × 100 (“DSE index”). Relations between DSE and, clinical, electrophysiologic parameters and the severity of tricuspid valve (TV) tethering (the RV length / tethering height during diastole: “Tethering index”) were assessed. In total patients, median DSE index and tethering index were 30.3 and 2.1 respectively, and the DSE index was correlated with tethering index (*r*_*s*_ = 0.664, *P* = 0.004). In 10 patients who underwent surgery, this association remained after surgery and at 2-year follow up. Tethering index ≥2.5 separated patients with and without persistent DSE. In conclusion, DSE exists in Ebstein’s anomaly. DSE index is related to the tethering index and DSE persists postoperatively if tethering index ≥ 2.5. As the persistent DSE might possibly impede the optimal recovery of RV function after surgery, severity of TV tethering should take into account in considering surgery.

## Introduction

Ebstein’s anomaly is a rare congenital disease with a wide spectrum of symptomatic statuses, from severely symptomatic and critical neonates to asymptomatic adults [1, 2]. Medical follow-up is recommended in asymptomatic patients, especially in children who have survived infancy without right-to-left shunt [1]. However, when there is evidence of deterioration, such as reduction in the right ventricular (RV) systolic function, increase in the RV size, and/or symptom progression, one should consider surgical correction [1]. Nevertheless, mainly due to the diverse outcomes, the practical indications for surgery have not been well established [2–6], and the operative indications should be considered on an individualized level.

After Carpentier et al. described the anatomic classification of Ebstein’s anomaly [7], diverse methods using echocardiography [4, 5, 8, 9] and cardiac magnetic resonance (CMR) images [10–12] have been suggested for assessing the severity of Ebstein’s anomaly and for predicting patient outcomes. These methods largely focus on the size of the functional RV and usually assess the ratio of the atrialized RV to the functional RV [4, 5, 8–12]. Parallel to this simple notion, the core of surgical correction is to incorporate the atrialized RV into functional RV, thereby increasing the size of functional RV. However, when considering the developmental characteristics of Ebstein’s anomaly, such as the partial absence of the myocardium [13–15], interstitial fibrosis of the septal wall [14, 16], and different electrical activities between the atrialized and functional RV [17, 18], simply incorporating the atrialized RV into functional RV might not be sufficient to normalize RV. Therefore, additional evaluation besides size is needed.

Dyssynchronous motion of the atrialized RV is occasionally observed in Ebstein’s anomaly; this has been described as paradoxical passive motion of the septal wall of the atrialized RV [1, 9, 14]. This physical characteristic frequently persists even after surgery when atrialized RV is incorporated into functional RV. This persisting dyssynchronous motion may probably result in suboptimal recovery of RV function, however, no study have focused on this abnormal motion of RV persisting even after surgery. Therefore, we intended to quantitatively assess dyssynchronous motion of the RV in Ebstein’s anomaly and to identify any preoperative factors correlated with the risk of persistent dyssynchronous motion after surgery.

## Methods

Between January 2006 and December 2016, 22 patients diagnosed with Ebstein’s anomaly in whom transthoracic echocardiography (TTE) and cardiac magnetic resonance (CMR) images were available at Seoul National University Hospital were initially selected. After exclusion of 5 patients due to (1) poor quality of TTE (n = 1), (2) combine other congenital disease (Tetralogy of Fallot, n = 1), (3) previous tricuspid valvular surgery (n = 2), and (4) disagreement of diagnosis between TTE and CMR (n = 1), 17 patients were retrospectively analyzed. Among 17 patients, 14 patients underwent corrective surgery. The surgical techniques used were Cone reconstruction, Danielson’s technique, other TV repair and TV replacement in 11, 1, 1, and 1 patients, respectively. After excluding 4 patients due to (1) poor quality of postoperative TTE (n = 1); (2) different operation methods, namely TV replacement (n = 1), Danielson technique (n = 1), and other TV repair (n = 1), 10 patients who underwent Cone reconstruction were analyzed for postoperative DSE analysis measured by acute postoperative TTE within 2 weeks. Among 10 patients, 8 patients were also available with 2-year follow up TTE and their DSE indexes were analyzed. This study was approved by the Institutional Review Board of Seoul National University Hospital (IRB No. 1702-025-829), which waived the need for informed consent, owing to the retrospective nature of the study.

All patients underwent a comprehensive echocardiographic examination using commercially available equipment (Vivid 7, GE Medical System, Horten, Norway). Acquisition of standard images and M-mode, two-dimensional, and Doppler measurements were performed in accordance with the guidelines provided by the American Society of Echocardiography [19, 20].

CMR images were obtained using a 1.5-T scanner (Magnetom Sonata or Magnetom Avanto, Siemens AG, Erlangen, Germany) equipped with adequate phased-array receiver coils under the standard protocols. The patients were scanned in the supine position. Steady-state free precession cine images were taken under a firm breath-hold to visualize both ventricular wall motions. All short-axis images were acquired at a 7-mm slice thickness with a 3-mm slice gap (Magnetom Sonata) or a 5-mm interval with a 5-mm interslice gap (Magnetom Avanto) from the base to the apex to include the whole ventricular volume.

Two new parameters were used in the analysis; Dyssynchronous Expansion Index (DSE index) and Tethering Index.

**Fig 1:**
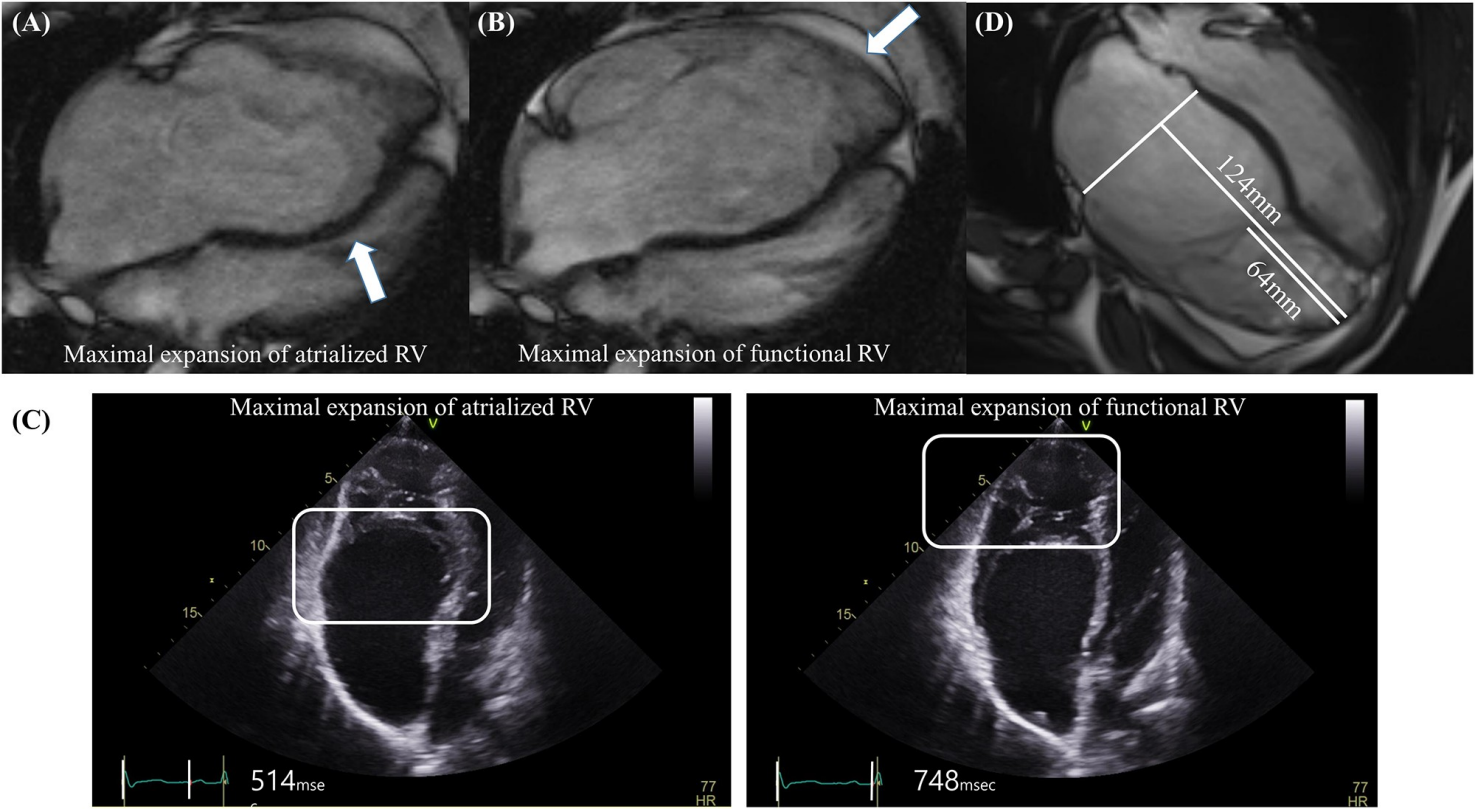
Explanation of diastolic dyssynchronous expansion (DSE) and methods used to calculate the DSE index and tethering index. (A) Maximal expansion of the atrialized right ventricle (arrow) occurs in early diastole. (B) In contrast, maximal expansion of the functional right ventricle (arrow) occurs in late diastole. (C) Measurement of the DSE index. DSE index = [{(time interval between QRS to maximal expansion of the functional RV)–(time interval between QRS to maximal expansion of the atrialized RV)}/ (cardiac cycle length)] × 100. For example, Q wave to peak expansion time of atrialized RV = 514 msec. Q wave to peak expansion time of functional RV = 748 msec. Cycle length = 779 msec. DSE index = ((748–514) / 779) x 100 = 30.0. (D) Measurement of the Tethering index. Tethering index = right ventricular length / tethering height. For example, right ventricular length = 124mm. Tethering height = 64mm. Tethering index = 124 mm/64 mm = 1.9.

1) DSE index: Thinned wall of the atrialized RV did not contract during systole. During early diastole, the thinned wall of the atrialized RV shows outward motion (Fig 1A), followed by rebound inward motion in late diastole when the functional RV was maximally expanded (Fig 1B) (S1 Video). We designated this abnormal diastolic motion as “diastolic dyssynchronous expansion (DSE) of the RV”. The severity of DSE was assessed by the time difference of maximal expansion between the atrialized RV and functional RV divided by the cardiac cycle length and calculated as follow: DSE index = [{(time interval between QRS to maximal expansion of the functional RV)–(time interval between QRS to maximal expansion of the atrialized RV)}/ (cardiac cycle length)] × 100 (Fig 1C). Although CMR can provide high quality images [21, 22], due to the limitation in the temporal resolution, TTE was used to estimate the DSE index. To minimize the effect of intraobserver variation, mean value of DSE indexes measured three times in different cardiac cycles was used for analysis.
2) Tethering index: The severity of TV tethering was calculated on CMR in the 4-chamber cine view: The tethering index = RV length / tethering height. The RV length was defined as the distance from the apex to the tricuspid annular plane at end-diastole. The tethering height was defined as the maximal distance from the RV apex to the distal leading edge of the TV leaflets during diastole (Fig 1D).

All continuous variables are presented as the mean ± standard deviation. Spearman correlation was used to analyze the linear correlations between parameters. Intraobserver and interobserver agreements for DSE index were analyzed using intraclass correlation coefficient. ICC estimate and its 95% confident intervals of intraobserver agreement were calculated based on a single-rating, absolute-agreement, 2-way mixed-effects model. ICC estimate and its 95% confidence intervals of interobserver agreement were calculated based on a mean-rating, consistency, 2-way mixed-effects model. A *P* value <0.05 was considered statistically significant. All statistical analyses were conducted using SPSS 20.0 (IBM Corp., Armonk, NY, USA). Graphs were drawn with GraphPad Prism 7.0 (GraphPad Software Inc., San Diego, CA, USA).

## Results

**Fig 2:**
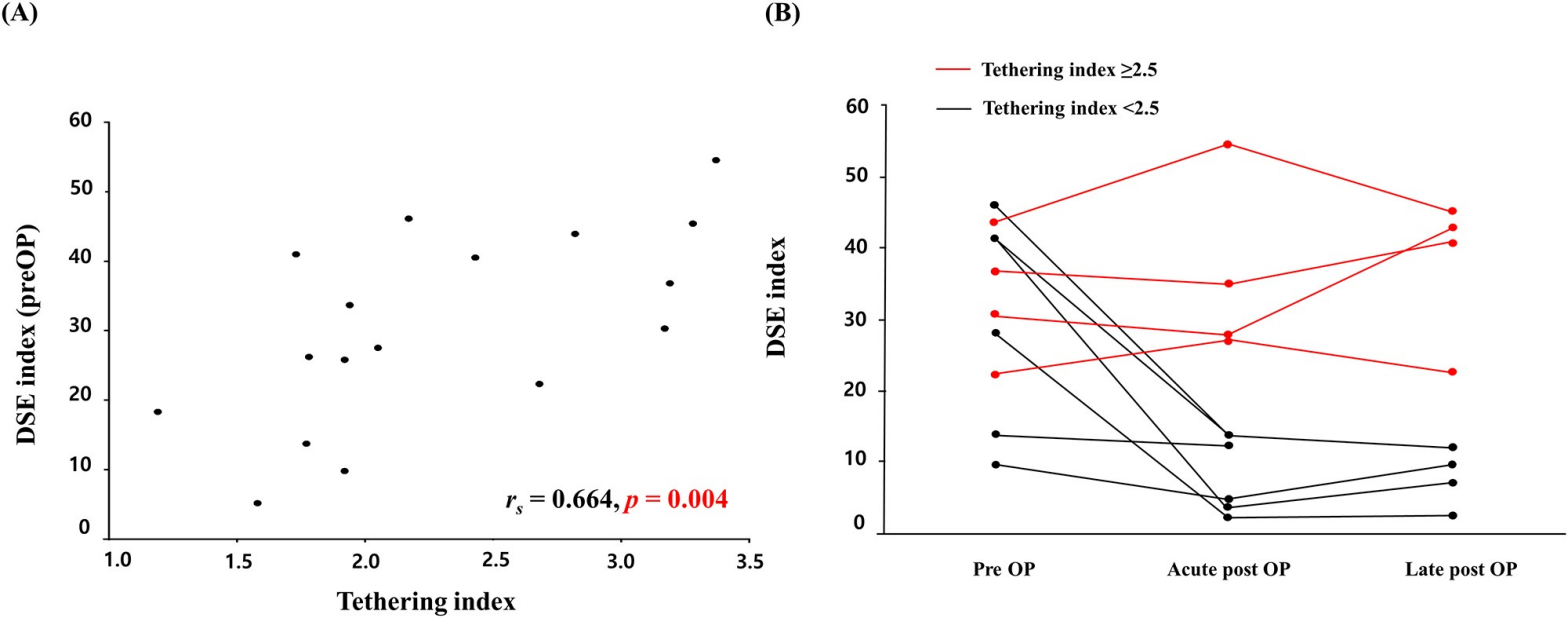
Association between diastolic dyssynchronous expansion (DSE) index and tethering index. (A) Association between DSE index and tethering index in total patients. (B) Postoperative changes in DSE index by tethering index 2.5.

The median age was 31 years (mean age 30.6 ± 4.4 years) and 11 (65%) were men. According to the Carpentier classification, 4, 12, and 1 patients were classified as types A, B, and C, respectively. Fifteen patients (88%) showed significant tricuspid regurgitation (> moderate degree). Mean DSE index of 17 patients was 30.7 ± 3.4 (range 5.2–54.6, median 30.3) and mean tethering index was 2.3 ± 0.2 (range 1.2–3.3, median 2.1) (Table 1). DSE index was correlated with tethering index (*r*_*s*_ = 0.664, *P* = 0.004) (Fig 2A) and with QRS duration (*r*_*s*_ = 0.485, *P* = 0.048), and tethering index showed higher correlation with DSE index (Table 2). Intraobserver agreement for DSE index was 0.954 (95.4%, 95% confidence interval, 0.901–0.981). Interobserver agreement for DSE index was 0.768 (76.8%, 95% confidence interval, 0.360–0.916).

**Table 1:** Baseline characteristics of total and postoperative patients.

	Total (n = 17)	PostOP patients (n = 10)
**Age, years**	30.6 ± 4.4	21.0 ± 4.4
**Men, n (%)**	11 (65)	6 (60)
**BSA, m**^**2**^	1.5 ± 0.9	1.4 ± 0.1
**BMI, kg/m**^**2**^	20.4 ± 0.8	19.0 ± 0.8
**Carpentier classification**		
**Type A, n (%)**	4 (24)	0 (0)
**Type B, n (%)**	12 (70)	9 (90)
**Type C, n (%)**	1 (6)	1 (10)
**Echocardiographic data**		
**LV ejection fraction, %**	67.8 ± 2.4	73.5 ± 2.5
**Tricuspid regurgitation**		
**Mild, n (%)**	2 (12)	2 (20)
**Moderate, n (%)**	4 (23)	2 (20)
**Severe, n (%)**	11 (65)	6 (60)
**DSE index**	30.7 ± 3.4	31.2 ± 4.0
**ECG parameters**		
**PR interval, ms**	184.0 ± 12.0	167.6 ± 9.6
**QRS duration, ms**	131.3 ± 6.5	138.4 ± 8.0
**QTc duration, ms**	463.2 ± 9.5	471.4 ± 11.5
**CMR images data**		
**RV length, mm**	94.0 ± 4.6	87.9 ± 5.8
**Tethering height, mm**	44.6 ± 3.7	39.2 ± 4.2
**Tethering index**	2.3 ± 0.2	2.4 ± 0.2
**PostOP DSE index values**		
**Acute postOP DSE index**		19.5 ± 16.6
**Late postOP DSE index**		22.9 ± 17.5

OP, operative; DSE, diastolic dyssynchronous expansion; CMR, cardiac magnetic resonance.

**Table 2:** Association between pre and postoperative DSE indexes and other parameters.

	Preoperative		Postoperative			
			Acute		Late	
	*r*_*s*_	*P* value	*r*_*s*_	*P* value	*r*_*s*_	*P* value
**Age**	0.173	0.507	-0.675	0.032	-0.539	0.168
**BSA**	0.130	0.619	-0.527	0.117	-0.548	0.160
**BMI**	-0.114	0.663	-0.729	0.017	-0.714	0.047
**Carpentier classification**	0.098	0.705	0.290	0.416	0.412	0.310
**Echocardiographic data**						
**LV ejection fraction**	0.158	0.544	0.413	0.235	0.443	0.272
**Tricuspid regurgitation grade**	0.104	0.691	-0.138	0.805	-0.069	0.872
**ECG parameters**						
**PR interval**	0.127	0.640	0.207	0.565	0.156	0.713
**QRS duration**	0.485	0.048	0.673	0.033	0.452	0.260
**QTc duration**	-0.023	0.929	0.164	0.650	0.084	0.844
**CMR images data**						
**Tethering index**	0.664	0.004	0.879	0.001	0.833	0.010

DSE, diastolic dyssynchronous expansion, CMR, cardiac magnetic resonance.

**Fig 3:**
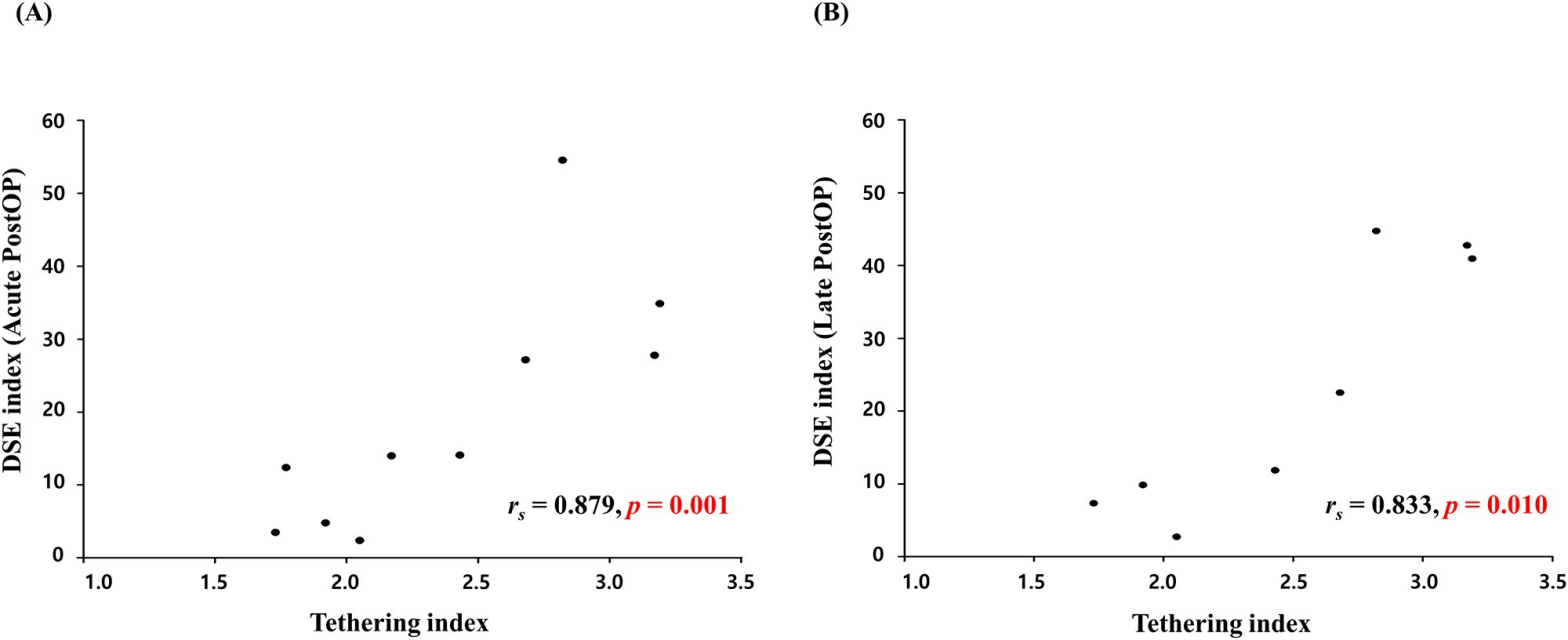
Association between the postoperative diastolic dyssynchronous expansion (DSE) index and preoperative tethering index. (A) Association between the acute postoperative DSE index and preoperative tethering index. (B) Association between the late postoperative DSE index and preoperative tethering index.

Ten patients underwent Cone reconstruction. According to the Carpentier classification, 0, 9, and 1 patients were classified as types A, B, and C, respectively, and 8 (80%) patients showed significant tricuspid regurgitation (> moderate degree) (Table 1). They were younger (median 16 years, mean 21.0 ± 4.4 years), had similar sex proportion (60% of men), greater preoperative DSE index (median 33.6, mean 31.2 ± 4.0), and preoperative tethering index (median 2.3, mean 2.4 ± 0.2) than total patients (Table 1). The younger (*r*_*s*_ = -0.675, *P* = 0.032), the longer QRS duration (*r*_*s*_ = 0.673, *P* = 0.033), and the greater preoperative tethering index (*r*_*s*_ = 0.879, *P* = 0.001) (Fig 3A) were correlated with acute postoperative DSE index (Table 2). Among the 10 patients, 2-year follow up TTE was available in 8 patients. Late postoperative DSE index was also correlated with preoperative tethering index (*r*_*s*_ = 0.833, *P* = 0.010) (Table 2) (Fig 3B).

**Fig 4:**
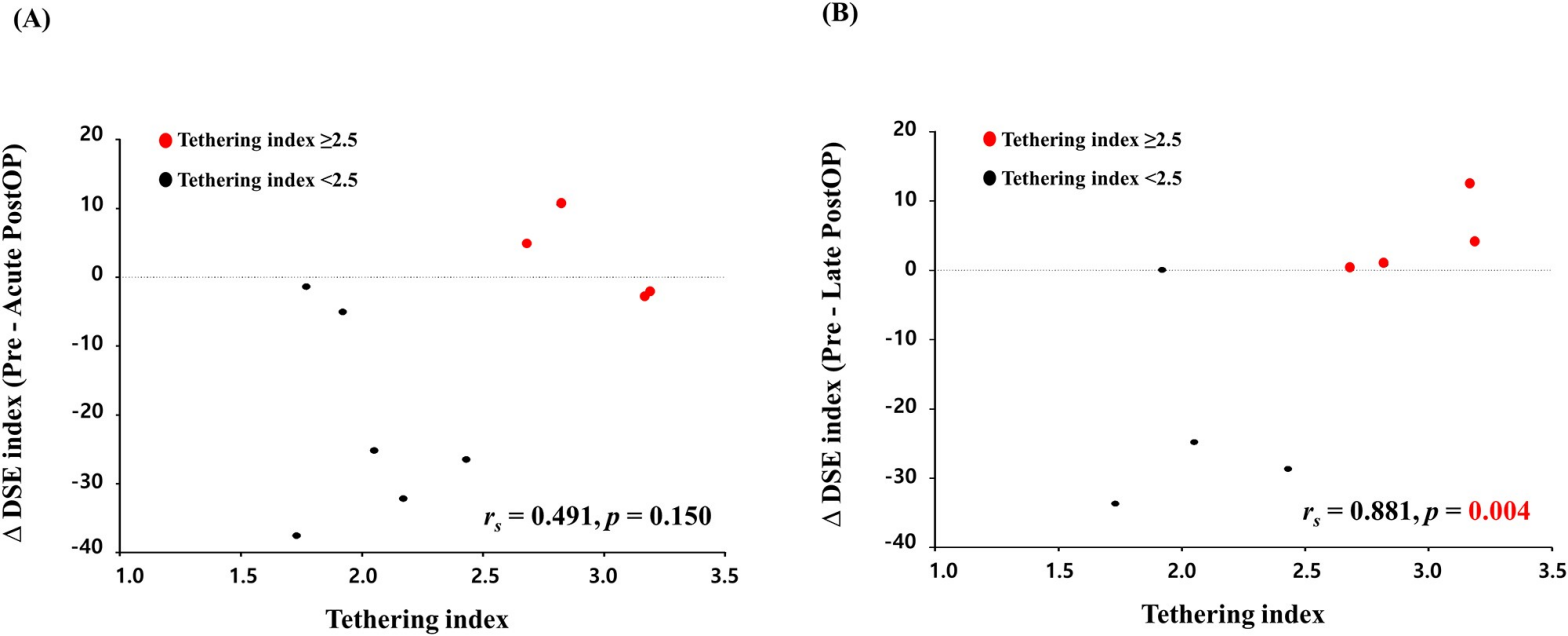
Association between the difference of pre and post diastolic dyssynchronous expansion (DSE) index and tethering index. (A) Association between the difference of pre and acute postoperative DSE index and preoperative tethering index. (B) Association between the difference of pre and late postoperative DSE index and preoperative tethering index.

The distribution of differences between pre- and postoperative DSE indexes showed different patterns according to the preoperative tethering index. Patients with a tethering index <2.5 tended to show an improved, or at least not aggravated postoperative DSE index. However, no patients with a tethering index ≥2.5 showed significant improvement of DSE after surgery (Figs 2B and 4A). The different pattern of postoperative DSE by tethering index 2.5 sustained at 2-year follow up TTE (Fig 4B). In this study, a preoperative tethering index of ≥2.5 was highly indicative of non-improvement of DSE after surgery.

## Discussion

Ebstein’s anomaly was first described in 1866 [23]. Although the natural and clinical courses have later been reported [23], these descriptions remain incomplete due to the relatively low frequency, and the wide variations in anatomic and hemodynamic profiles [4]. Symptomatic neonate patients have a poor prognosis and high mortality and there are patients showing progressive right-sided heart failure in childhood [1, 24]. However, patients who reach adulthood have excellent outcomes [1, 5, 25] and, in these patients, decision-making for surgery should be more considerate and should be individualized.

We found that the atrialized RV showed remarkably different movements compared to the functional RV, which appears as a dyssynchronous RV movement (S1 Video). Previously, this unique movement have been recognized as paradoxical passive septal motion [1, 9], but no quantified approaches have been applied. In one study, synchrony between the atrialized and functional RV was assessed with cineangiography by relative volume changes [17]. This study concentrated only on the synchrony in systole, however, if we look for the synchrony in diastole from the data presented, timing of maximal expansion between atrialized and functional RV was quite opposite to our finding. However, the frame-by-frame analysis of cineangiography may have intrinsic limitations in the analysis of synchrony in two chambers. Regardless of the contradictory results, this study supports the fact that the atrialized RV shows dyssynchronous movement to the functional RV during diastole.

Despite the recent advance in the evaluation of RV function, it is still practically impossible to suggest numerical data regarding incomplete restoration of RV function because of the diastolic dyssynchrony even after the optimal restoration of functional RV size. However, it is quite reasonable to assume that diastolic dyssynchrony might result in ineffective diastolic filling and therefore, incomplete restoration of RV function expected for the restoration of RV cavity size.

In our study, severity of TV tethering affects the degree of residual DSE and the greater the preoperative tethering index, the higher the postoperative DSE index. Based on these findings, we concluded that less optimal outcome might be predicted in patients with tethering index greater than 2.5.

This study has some limitations. First, the study was limited by the small number of patient included. However, considering the low prevalence of Ebstein’s anomaly and its surgical correction, our findings may still provide new insight into the selection of patients with Ebstein’s anomaly for surgery. Second, this is a retrospective study, and the outcome and mortality information was limited. Further large-scale prospective studies are required to address these issues and to confirm our findings.

In summary, DSE exists in Ebstein’s anomaly and DSE may persist even after surgery. We noticed that postoperative DSE is related to the severity of TV tethering preoperatively. Presence of the DSE postoperatively, may impede the full recovery of RV function expected from the restoration of functional RV size. Therefore, severity of TV tethering should be taken into account in considering surgery in patient with Ebstein’s anomaly.

## Supporting information

S1 Data All relevant data are within the supporting information file. (XLSX)

S1 Video Representative case of diastolic dyssynchronous expansion (DSE) of the right ventricle. Tethering index was 3.2. DSE of this patient was not improved (DSE index, 30.3 to 27.7).
(AVI)
